# Separation Mechanisms and Anti-Fouling Properties of a Microporous Polyvinylidene Fluoride–Polyacrylic Acid–Graphene Oxide (PVDF-PAA-GO) Composite Membrane with Salt and Protein Solutions

**DOI:** 10.3390/membranes13010040

**Published:** 2022-12-28

**Authors:** Li-Ting Wang, Yu-Han Chen, Wei-Ting Chang, Selvaraj Rajesh Kumar, Chien-Chang Chen, Shingjiang Jessie Lue

**Affiliations:** 1 Department of Chemical and Materials Engineering, Chang Gung University, Taoyuan City 333, Taiwan; 2 Division of Pediatric Gastroenterology and Hepatology, Department of Pediatrics, Chang Gung Memorial Hospital, Taoyuan 333, Taiwan; 3 College of Medicine, Chang Gung University, Taoyuan 333, Taiwan; 4 Department of Orthopedics, Chang Gung Memorial Hospital, Linkou, Taoyuan City 333, Taiwan; 5 Department of Safety, Health and Environment Engineering, Ming Chi University of Technology, New Taipei City 243, Taiwan

**Keywords:** polymer–graphene oxide composite, microporous membrane, protein separation, anti-fouling

## Abstract

This research demonstrates the preparation of composite membranes containing graphene oxide (GO) and investigates the separation mechanisms of various salts and bovine serum albumin (BSA) solutions. A microporous polyvinylidene fluoride–polyacrylic acid–GO (PVDF-PAA-GO) separation layer was fabricated on non-woven support. The GO-incorporating composite resulted in enlarged pore size (0.16 μm) compared with the control membrane (0.12 μm). The zeta potential of the GO composite was reduced to –31 from –19 mV. The resulting membranes with and without GO were examined for water permeability and rejection efficiency with single salt and BSA solutions. Using the non-woven/PVDF-PAA composite, the permeance values were 88–190 kg/m^2^hMPa, and the salt rejection coefficients were 9–28% for Na_2_SO_4_, MgCl_2_, MgSO_4_, and NaCl solutions. These salt removals were based on the Donnan exclusion mechanism considering the ion radii and membrane pore size. Incorporating GO into the separation layer exhibited limited impacts on the filtration of salt solutions, but significantly reduced BSA membrane adhesion and increased permeance. The negatively charged protein reached almost complete removal (98.4%) from the highly negatively charged GO-containing membrane. The GO additive improved the anti-fouling property of the composite membrane and enhanced BSA separation from the salt solution.

## 1. Introduction

The demand for limited water resources is increasing due to the growing population of the world [1]. Desalination technologies, including membrane and thermal processes, offer the solutions to generating fresh water from salty water. Membrane technology is based on thin films (with either porous or dense structure) for the retention of certain molecules (such as viruses, bacteria, proteins, metals, and salts) while allowing water molecules to pass through. In these systems, less energy is required, and they have lower costs than other conventional processes [2]. Ultrafiltration (UF), reverse osmosis (RO), microfiltration (MF), forward osmosis (FO), electrodialysis (ED), and nanofiltration (NF) [3] are commonly applied for aqueous streams. Among these, UF is a pressure-driven membrane process with a pore size distribution of 0.001–0.1 μm (or molecular weight cut-off of 1000–1,000,000 Daltons), which can reject suspended solids, bacteria, macromolecules, etc. [4,5]. Usually, it is operated with a pressure of 1–10 bar and generates higher permeate flux at a lower cost than RO and NF processes [4,5]. 

The separation principle in a UF process is mainly a sieving mechanism, where solute rejection is determined by solute size and shape relative to the membrane pores [6]. However, it is proposed that the membrane surface charge also affects the salt removal efficiency due to electrostatic interaction [7]. These attractive advantages enable UF to become a significant piece of technology in various applications, such as pretreatment prior to the RO process, industrial wastewater treatment [8], hemodialysis [9], food processing [10], and biomolecule separation [11]. For instance, enzymes, antibodies, protein drugs, and valuable macromolecules have to be separated from solutions to reach purity requirements [12]. 

Among many polymer materials, polyvinylidene fluoride (PVDF) membranes are extensively used for the UF method in water treatment owing to PVDF’s outstanding chemical and oxidation resistance, high thermal stability, film-forming property, and desirable mechanical properties [13,14]. However, its hydrophobic nature attracts proteins and other particles to block the membrane’s surface [15]. Some methods have been proposed to improve the hydrophilicity and anti-fouling of PVDF membranes [16,17]. For instance, PVDF blended in a PVDF–polyacryloylmorpholine (PACMO) copolymer and polyvinylpyrrolidone (PVP) exhibited better permeability performance, but poorer BSA rejection, than a pristine PVDF membrane [18]. In our previous works, we mixed polyacrylic acid (PAA) or polyvinyl pyrrolidone (PVP) into the PVDF matrix to increase the hydrophilicity and negative zeta potential to improve the permeance and salt/dye rejection efficiencies [19,20].

Two-dimensional graphene oxide (GO) is derived from exfoliated oxidized graphite and has a high specific surface area and good mechanical strength [21,22]. The oxygen-containing structure of GO can increase the hydrophilicity in the PVDF composite membrane [20], thereby improving the membrane filtration and fouling resistance [23]. In our previous report, GO nanosheets were deposited on PVDF/PAA polymeric support, and the interlayer spacing of GO could be fine-tuned to reject salts. The permeance, however, was decreased by 80–95.5% from the level of the GO-free membrane [20]. Later we blended the GO with the polyvinyl pyrrolidone (PVP) and PVDF mixture to improve the polymeric chemical compatibility and increase the porous structure [19]. This porous PVDF/PVP-GO composite membrane included two- to four-fold increases in dye rejections, while maintaining a higher permeance than that of the GO-free membrane. 

In this research, we propose the direct blending of GO into a PVDF-PAA solution and casting that on a non-woven support to form a microporous separation layer via the phase inversion method. The as-synthesized GO suspension solution was redistributed in an organic solvent and directly mixed with a PVDF-PAA polymer solution without being dried into a powder. The cast film solidified and formed a microporous structure upon immersion in water as a result of solvent exchange. The direct blending of GO in the composite increased the pore size, hydrophilicity, and volume porosity as compared with the pristine sample. The objectives were to investigate the permeance and the separation mechanism with respect to protein or various single salt solutions. The filtration performance and the anti-fouling effects of the non-woven/PVDF-PAA-GO composite were compared with those of a non-woven/PVDF-PAA membrane.

## 2. Materials and Methods

### 2.1. Materials

Poly(acrylic acid) (PAA), poly(vinylidene fluoride) (PVDF, MW = 534,000 Da), graphite, sodium chloride (NaCl), N-methyl-2-pyrrolidinone (NMP) solution, sulfuric acid (H_2_SO_4_) (95–98%) solution, and bovine serum albumin (BSA) were acquired from Sigma-Aldrich, St. Louis, MO, USA. Magnesium sulfate (MgSO_4_), decahydrate sodium sulfate (Na_2_SO_4_·10H_2_O), hexahydrate magnesium chloride (MgCl_2_·6H_2_O), and potassium permanganate (KMnO_4_) chemicals were obtained from Showa Chemical Inc. and Nihon Shiyaku Industries, Ltd., Osaka, Japan. The non-woven starter solution (model 3706, prepared from polyethylene terephthalate) was acquired from Ahlstrom Corp., Manchester, UK.

### 2.2. Preparation and Testing of the Composite Membrane

#### 2.2.1. Preparation of GO

The modified Hummer routine was applied to prepare GO with slight modifications [24]. Initially, the graphite powder (400 mg) was added to 60 mL of 98% H_2_SO_4_ solution at ambient temperature with 10 min continuous mechanical stirring at 200 rpm. Next, 400 mg of KMnO_4_ (1 wt%) powder was directly added to the solution. After 30 min of continuous stirring, again, we mixed in more KMnO_4_ (total of 5 wt%) until the green MnO^3−^ color diminished. Once the reaction was complete, the ice cubes (80 g) were mixed with the above solution [20]. This experiment was placed in an ice bath, resulting in the colloidal solution displaying a purple color. The colloidal solution was left overnight at room temperature. The black precipitate was separated from the solution and washed several times with deionized (DI) water until the pH of the precipitated solution became neutral [20]. The GO aqueous solution was centrifuged, and the water was replaced with NMP solvent [25] to redistribute GO in the NMP solution.

#### 2.2.2. Fabrication of the Non-Woven/PVDF-PAA Membrane

The PVDF-PAA copolymer solution was synthesized by mixing PVDF powder (6 g) and PAA (0.12 g) powder into 24 g of NMP and heated at 60 °C with continuous stirring for 12 h. The mixture was left for another 12 h to allow bubbles to be released, and the polymer solution was cast onto non-woven support with an adjustable film applicator (with 200 μm knife blade clearance and 15 mm/s of a cast speed). The non-woven membrane containing the cast PVDF-PAA solution was immersed in DI water at room temperature for the phase inversion to occur. The resulting composite membrane was placed in a vacuum oven and dried at 60 °C [20].

#### 2.2.3. Fabrication of the Non-Woven/PVDF-PAA-GO Membrane

The suspension of GO (3 mg) in NMP was added into the PVDF-PAA solution, as aforementioned, to obtain a homogeneous slurry. The PVDF-PAA-GO solution was cast on non-woven support, immersed in DI water, and placed in a vacuum oven at 60 °C to obtain the dried composite membrane.

### 2.3. Material and Membrane Characterizations

#### 2.3.1. Physicochemical Properties of GO

A transmittance electron microscope (HRTEM, JEM 2010, JEOL, Tokyo, Japan) was used to observe the GO microstructure. The functional groups of the GO were evaluated using Fourier-transform infrared spectroscopy (FTIR) (FT-730, Horiba, Ltd., Kyoto, Japan) after being mixed with KBr (1:300, *w*/*w*) and pressed into a pellet. The chemical composition of the GO was studied using X-ray photoelectron spectroscopy (XPS, VG Microtech MT-500 ESCA, Thermo Fisher, Waltham, MA, USA). The D and G band ratios of the GO were observed using Raman spectroscopy (UniDRON, UniNanoTech, Seoul, Korea). The GO suspension was dried and analyzed using an X-ray diffraction analyzer (XRD, model D5005D, Simens AG, Munich, Germany) with a scan range of 2θ from 5° to 70°. The light source was Cu Kα radiation with a wavelength of 1.54056 Å applied at a voltage of 40 kV and a current of 40 mA. The GO mass loss at elevated temperatures was analyzed using a thermogravimetric analyzer (TGA, Q50, TA Instruments, New Castle, DE, USA) in an air atmosphere and heated at a rate of 10 °C/min. The zeta potential and size distribution of GO solution (50 ppm) in various pH solutions (using 1 M NH_3_ and 0.1 M HCl solutions) were studied using a laser light scattering analyzer (Zetasizer, 2000HAS, Malvern, Worcestershire, UK). The zeta size and potential of the BSA solution (500 ppm) in the neutral aqueous solution were also determined using the Malvern laser light scattering analyzer.

#### 2.3.2. Physicochemical Analysis of the Composite Membrane

The composite membrane sample was dried well and then cryo-fractured by liquid nitrogen so the cross-sectional micrograph could be studied. Initially, the sample was sputtered with gold, and the microstructure of the membranes was analyzed by field emission scanning electron microscopy (FESEM, SU8820, Hitachi, Tokyo, Japan). The mean pore size distributions of the composite membranes were studied using a capillary flow porometer (CFP-1500-AE, Porous Materials Inc., Ithaca, NY, USA). The gravimetric method was used to calculate the volume porosity [19]. The hydrophobic/hydrophilic properties of the composites were analyzed using a contact angle (G10, Kruss GmbH, Hamburg, Germany). The surface zeta potential of samples was determined by Otsuka ELSZ-2000 (Otsuka Electronics Co., Ltd., Osaka, Japan).

### 2.4. Membrane Filtration Performances

#### 2.4.1. Preparation of Feed Solutions and Calibration Curves

About 2 g of salt was mixed with 1 L of DI water. The resulting concentrations were 2000 ppm for NaCl and MgSO_4_, 938 ppm for MgCl_2_, and 883 ppm for Na_2_SO_4_. The standard solutions were further diluted, and the calibration curves of different salt solutions (NaCl, MgCl_2_, Na_2_SO_4_, and MgSO_4_) were obtained using a conductivity meter for concentration measurement, as represented in the supplementary information (Figure S1a–d). The BSA solution was prepared by mixing 0.5 g of BSA into 1 L of DI water. The concentration was quantified using a UV/Vis spectrometer (V-650, JASCO, Tokyo, Japan) at a wavelength of 280 nm, and the diluted standards’ calibration curve was obtained (Figure S1e).

#### 2.4.2. Membrane Filtration Performance Test

The tested membrane (effective area of 4.9 × 10^−4^ m^2^) was positioned into a filtration test unit with recycling mode. Salt or BSA feed solution at 25 °C was fed as a crossflow into the module at a pressure of 0.49 MPa. For the water-only and salty solutions, the permeate flow rates and salt concentrations were measured every 20 min using a flow meter and conductivity meter, respectively. For 500 ppm BSA solutions, the data were recorded after 3, 6, 12, 24, 48, 72, 96, and 120 h of operation. The BSA concentration was studied using a UV–Vis spectrophotometer. The water permeance and rejection coefficient were calculated according to the literature [5,20]. The flux recovery ratio was calculated as the ratio of recovered flux after the water rinse protocol according to the literature [19]. 

## 3. Results and Discussion

**Figure 1:**
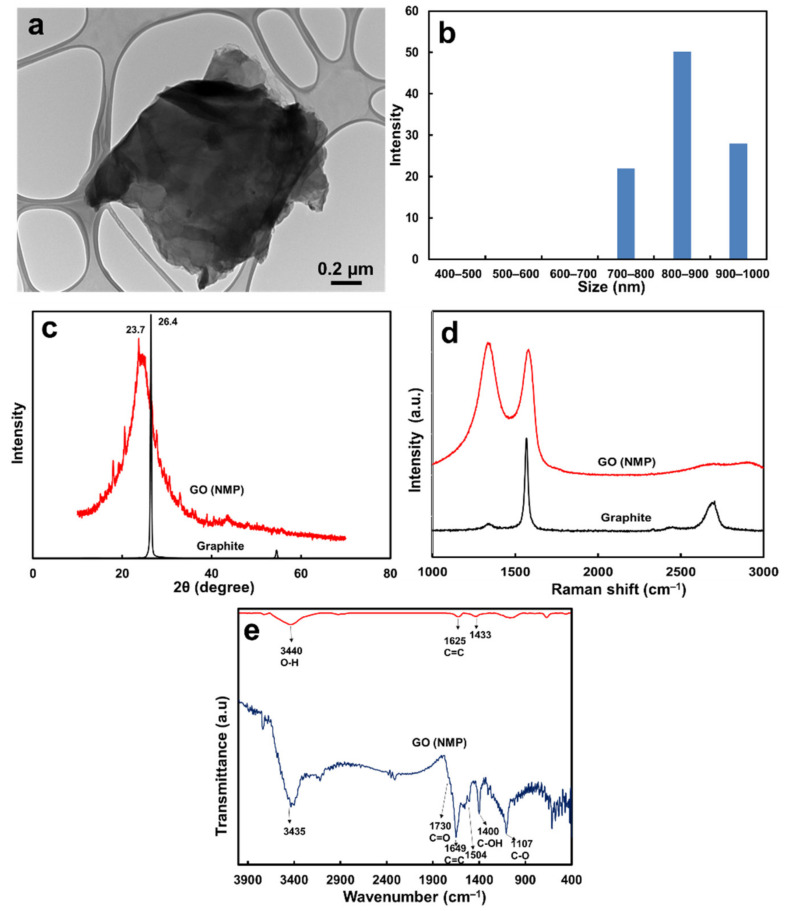
(**a**) TEM micrograph and (**b**) particle size distribution of GO in NMP solution, (**c**) XRD patterns, (**d**) Raman spectra, and (**e**) FTIR spectra of commercial graphite and GO prepared from NMP solution.

The GO morphology was observed using TEM. The GO distributed in the NMP solvent showed a flaky structure with wavy wrinkles, as represented in Figure 1a. However, the GO nanosheets tended to be stacked into multilayers, unlike the mono-dispersed GO in water (Figure S2a) [25]. The GO in organic solvent might have lacked molecules to form interlayer hydrogen bonds [26] and tended to stack into a larger lamellar structure than in the aqueous solution. These GO lamellar stacks ranged from 0.7 to 1.0 μm in diameter (Figure 1b) and, in the aqueous solution, ranged from 0.2 to 0.5 μm (Figure S2b).

The d-spacing values of the commercial graphite powder, GO in the aqueous solution, and GO redistributed in the NMP solution were evaluated using XRD. The commercial graphite powder showed a diffraction peak (002) at 2θ = 26.4° (Figure 1c), corresponding to a d-spacing of 3.4 Å (0.34 nm) using Bragg’s equation. After the commercial graphite was oxidized using the modified Hummer technique, the GO d-spacing in an aqueous solution was enlarged to 7.98 Å (0.798 nm) [20], as shown in Figure S2c. The oxygen-containing functional groups (including epoxy, hydroxyl, carboxyl, and carbonyl groups) and intercalated water molecules formed hydrogen bonds and increased the d-spacing [20,27,28]. When the GO was redistributed from an aqueous into an NMP solution, the diffraction peak at 2θ of 23.7° corresponded to a d-spacing of 3.75 Å (0.375 nm, Figure 1c). The GO lamellar size was larger (0.7 to 1 μm) in the NMP solution than in the aqueous solution (0.2 to 0.5 μm) [25]. From these results, it can be inferred that the redistribution of GO in the NMP caused originally exfoliated GO single layers in the aqueous solution to form aggregates or a lamellar structure, and the d-spacing shrunk due to π–π stacking and hydrophobic interaction of the C–C bonds. The carbon bond structures of the graphite and GO were analyzed using Raman spectroscopy, as shown in Figure 1d, the graphite had a high intensity of the G band (~1569 cm^−1^) with an intensity ratio (I_D_/I_G_) of 0.28, and the ratio of the GO dried from its NMP solution was 1.03 (Figure 1d). The aqueous GO exhibited an I_D_/I_G_ ratio of 1.00 [25], as represented in Figure S2d. This indicates that the GO redistributed in the NMP remained in its carbon bond structure; both GO in the aqueous solution and GO redistributed in the NMP solution exhibited half of the carbon bonds of the planar sp^2^ structure and half of the sp^3^ structure [20,27]. Moreover, the G band of GO in the NMP solution shifted to a higher wavenumber, which implied fewer layers than the pristine graphite sample [29,30]. 

The functional groups of the graphite and GO prepared from the NMP solution were studied using FTIR spectroscopy, and the results are shown in Figure 1e. Only a few peaks were observed in the graphite sample: the bands at 3440 cm^−1^ corresponding to O–H stretching vibration from the small amount of adsorbed water molecules, the band at 1625 cm^−1^ corresponding to C=C stretching vibration, and that at 1433 cm^−1^, attributed to the skeletal vibration of the graphite sheets [31,32,33,34,35]. In the NMP-solvated GO spectra, the bands at 1107 and 1730 cm^−1^ were attributed to the stretching vibrations of the C–O (hydroxyl) and C=O (carboxyl) groups, which confirmed the GO formation [26]. The bands at 1504 and 1649 cm^−1^ were associated with C=C groups. The bands at 1400 and 3435 cm^−1^ were for the C–OH and O–H groups, respectively. The GO in an aqueous solution also showed a similar FTIR peak position, which confirmed the successful formation of GO, as displayed in Figure S2e. 

### 3.1. Characterization of Polymeric Composite Membranes

**Figure 2:**
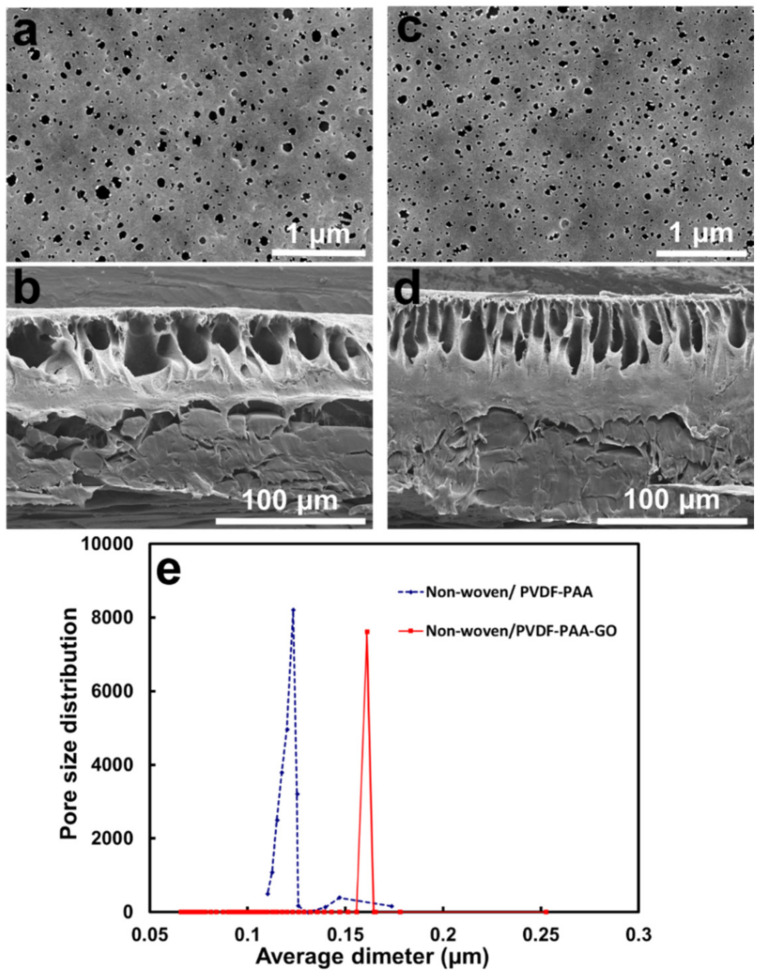
Microstructure of (**a**) top surface and (**b**) cross-section of non-woven/PVDF-PAA composite. (**c**) Top surface and (**d**) cross-section of non-woven/PVDF-PAA-GO composite. (**e**) shows the pore size distribution of non-woven/PVDF-PAA and non-woven/PVDF-PAA-GO membranes.

The non-woven support had a thickness of nearly 100 μm and a pore size of 7.13 ± 3.33 μm. Its zeta potential was almost neutral (−3.91 mV), and the contact angle was 77.2° (Table 1). The cast PVDF-PAA was 56 μm thick. The surface layer revealed a porous morphology, and the cross-section had a finger-like structure (Figure 2a,b). The mean pore size distribution was 0.12 μm in diameter. The volume porosity was 15.6%. The water contact angle on the cast PVDF-PAA membrane was 74.9° (Table 1), which was lower than that of the pure PVDF porous membrane (79.2°) due to the increased hydrophilicity caused by the PAA incorporation. The PAA grafted on polymeric membranes could improve the hydrophilicity and convert neutral membranes to charged membranes [20,36,37,38]. The small amount of PAA additive also contributed to a lower surface zeta potential of −19.36 mV, which was more negative than that of the non-woven membrane (−3.91 mV). The weakly anionic polyelectrolyte PAA was deprotonated and formed carboxylate functional groups, resulting in a negatively charged polymeric surface.

When 0.05% GO was mixed with the PVDF-PAA polymer solution, the resulting PVDF-PAA-GO layer on top of the non-woven membrane revealed a similar thickness (Table 1) as that of the control PVDF-PAA membrane. The surface layer revealed a narrower pore size distribution, and the cross-section had a denser finger-like structure (Figure 2c,d) than that without the GO composite film. The mean pore size (0.16 μm) and volume porosity (28.7%) were slightly larger than those without GO addition (Figure 2e, Table 1). The hydrophilicity of GO accelerated the interchange of non-solvent/solvent during the polymer phase inversion process [39], resulting in increased pore size of the membrane. Safarpour et al. found that the mean pore size of the top layer and porosity of PVDF were 0.55 μm and 0.73, whereas the GO and rGO/TiO_2_ additions to the composite films enlarged the pores to 0.7–0.76 μm, and the porosity was 0.82–0.83 [17]. Wang et al. observed a similar structure in PVDF-GO membranes when GO content was less than 0.1 wt% [16]. They also reported that the average mean pore size of the membrane increased as the content of GO was increased when GO was less than 0.2 wt% [16]. Our pore enlargement upon GO addition into the PVDF-PAA matrix was consistent with the reported literature.

Moreover, the water contact angle of the non-woven/PVDF-PAA-GO membrane was decreased to 59.3° (Table 1), which was due to the presence of the hydrophilic GO nanosheets [16]. Moreover, the surface zeta potential of the PVDF-PAA-GO composite was more negatively charged (−30.88 mV) than PVDF-PAA (−19.36 mV), being contributed by GO (with a zeta potential of −33 mV [20]), as represented in Table 1.

### 3.2. Performance of Non-Woven/PVDF-PAA Membrane

#### 3.2.1. Permeance and Separation Performance on Salt Solutions

The pure water permeance was first determined on a non-woven/PVDF-PAA composite membrane. The permeance value of pure water was 246 kg/m^2^hMPa, which is higher than or comparable with literature values [20,37,40], as described in Table 2. 

**Figure 3:**
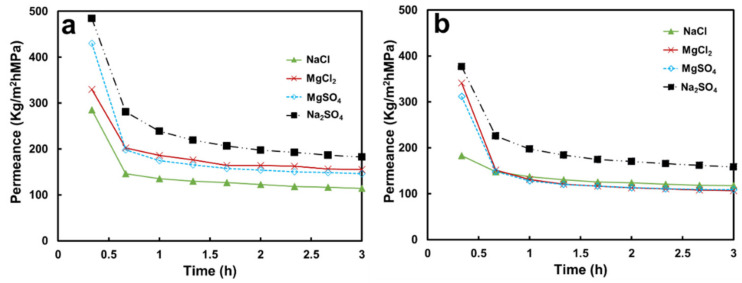
Time-resolved water permeance values for various salt solutions using (**a**) a non-woven/PVDF-PAA membrane and (**b**) non-woven/PVDF-PAA-GO composite membranes. Salt solutions: 2000 ppm for NaCl and MgSO_4_, 938 ppm for MgCl_2_, and 883 ppm for Na_2_SO_4_.

**Figure 4:**
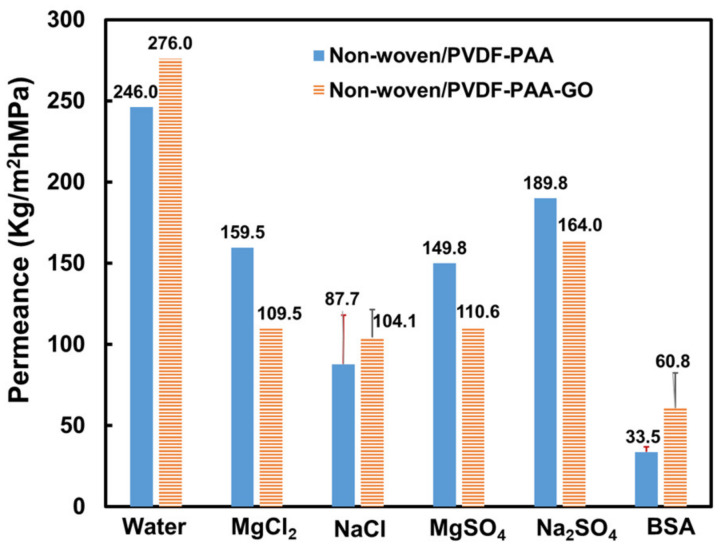
Steady-state water permeation of salt and BSA solutions using non-woven/PVDF-PAA and non-woven/PVDF-PAA-GO composite membranes. Salt solutions: 2000 ppm for NaCl and MgSO_4_, 938 ppm for MgCl_2_, and 883 ppm for Na_2_SO_4_; BSA solution: 500 ppm. The values of NaCl and BSA are the mean ± 95% confidence intervals based on two replicates (n = 2).

The permeance values of non-woven/PVDF-PAA composite declined with operating time for the four tested salt solutions (Figure 3a) as compared with pure water permeance. The Na_2_SO_4_ solution had the highest permeance of 189.8 Kg/m^2^hMPa, whereas MgCl_2_, MgSO_4_, and NaCl solutions resulted in reduced permeances of 159.5, 149.8, and 87.7 Kg/m^2^hMPa, respectively (Figure 4). These values are comparable with those in the literature [20] after the experimental errors were taken into consideration. 

The water permeance is controlled by the driving force—water activity—of the filtration process [5]. When pure water is the feed solution, the water activity has unity. The high driving force resulted in the high permeance (246 kg/m^2^hMPa) through the non-woven/PVDF-PAA membrane. When the water was replaced with the salt solution, the water activity was inevitably reduced due to the presence of the salt solute. The permeance from the salty solution was lowered as well. The calculated salt activity was in the order of NaCl (0.028) > MgSO_4_ (0.025) > MgCl_2_ (0.021) > Na_2_SO_4_ (0.014), as shown in Table 3. A similar trend was found for the osmotic pressure exerted by the salt solutions. Then, the water activity was ranked in the reverse order, and the permeance values were expected to be NaCl < MgSO_4_ < MgCl_2_ < Na_2_SO_4_. This prediction is in line with our experimental data (Figure 4).

**Figure 5:**
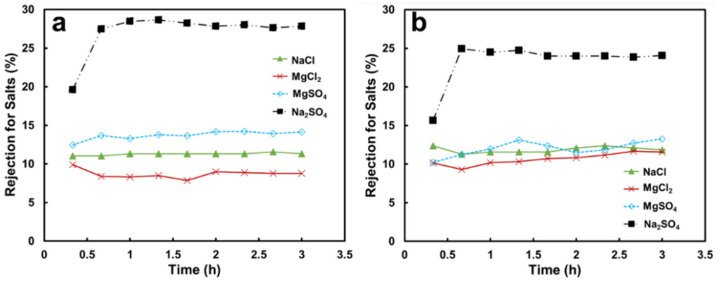
Time-resolved salt rejection coefficient using (**a**) non-woven/PVDF-PAA and (**b**) non-woven/PVDF-PAA-GO composite membranes. Salt solutions: 2000 ppm for NaCl and MgSO_4_, 938 ppm for MgCl_2_, and 883 ppm for Na_2_SO_4_.

**Figure 6:**
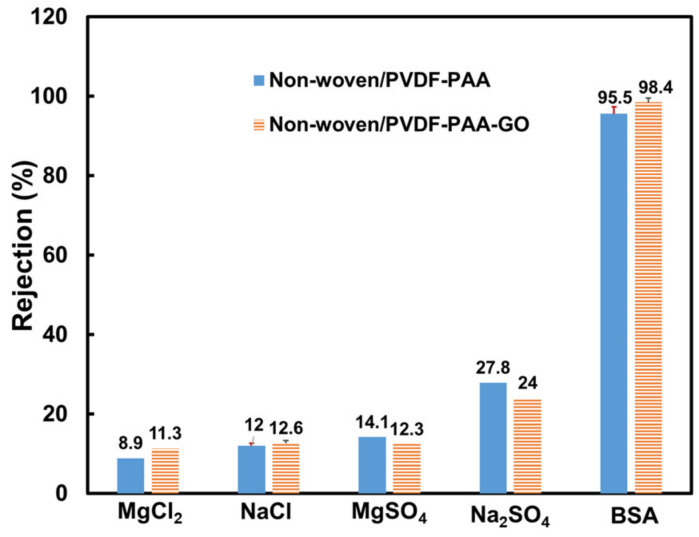
Steady salt rejection coefficient for salt and BSA solutions on non-woven/PVDF-PAA and non-woven/PVDF-PAA-GO composite membranes. Salt solutions: 2000 ppm for NaCl and MgSO_4_, 938 ppm for MgCl_2_, and 883 ppm for Na_2_SO_4_; BSA solution: 500 ppm. The error bars in NaCl and BSA are 95% confidence intervals based on two replicates (n = 2).

The associated ion hydrated radii were 0.36 and 0.43 nm for Na^+^ and Mg^2+^ and 0.30 and 0.32 nm for SO_4_^2−^ and Cl^−^, respectively [41]. Such small salts (size in the <1 nm range) were not expected to demonstrate any removal using the non-woven/PVDF-PAA composite (with an average pore size of 0.12 μm). It is interesting to note that Na_2_SO_4_ was rejected at 27.8%, MgSO_4_ at 14.1%, NaCl at 12%, MgCl_2_ at 8.9%, and BSA at 95.5% (Figure 5 and Figure 6). The rejection coefficients of the sulfate solutions were higher than those of the chloride solutions due to the Donnan exclusion mechanism or electrostatic repulsion [42]. The non-woven/PVDF-PAA composite membrane contained 2% PAA, and the resulting membrane was negatively charged (Table 1). While in contact with the negatively charged PVDF-PAA, the larger charge density of the sulfate ions exerted a more intensive repulsive force than the chloride ions. Therefore, sulfate solutions were removed at higher rates than Cl^−^ solutions, and this result is consistent with literature findings with sodium and calcium cations at 0.001–0.01 M [42,43]. It is assumed that the membrane tends to exclude co-ions and associated counter-ions to maintain solution electro-neutrality in the permeate (downstream of the composite membrane).

#### 3.2.2. Separation Performance on BSA Solution

**Figure 7:**
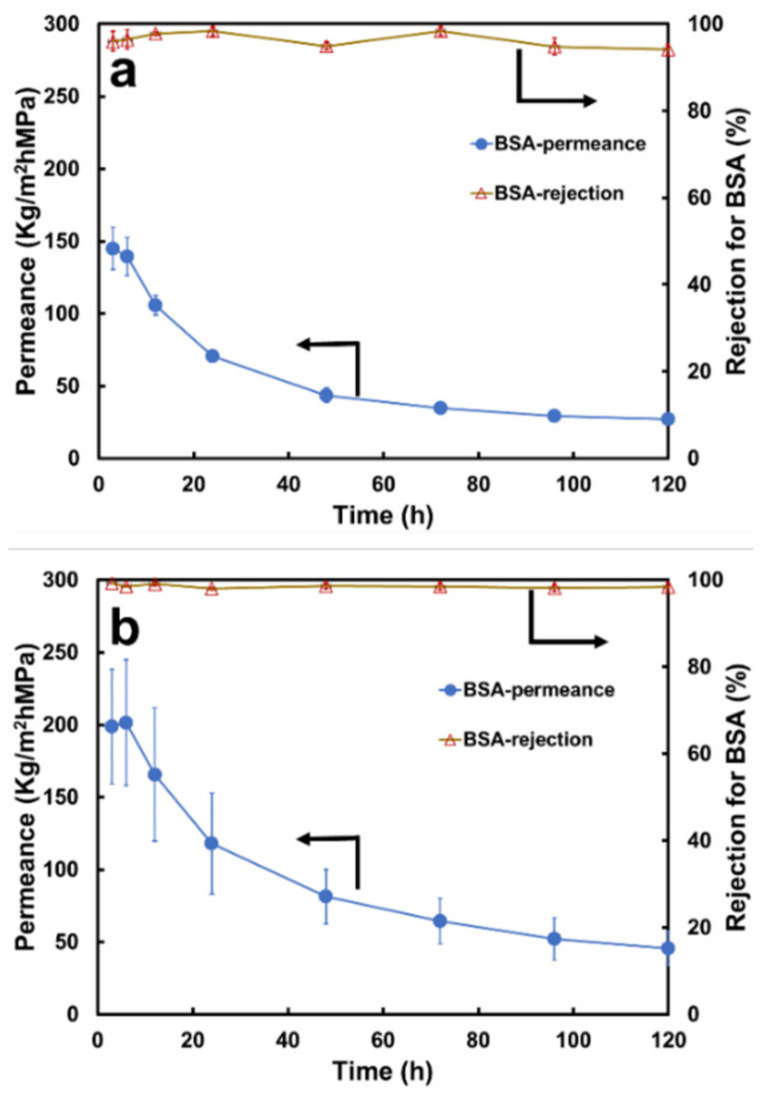
Permeance and rejection of BSA solution using (**a**) non-woven/PVDF-PAA and (**b**) non-woven/PVDF-PAA-GO membranes. The error bars are 95% confidence intervals based on two replicates (n = 2).

The permeance of the BSA solution declined with operating time (Figure 7a). The initial value was 144.84 ± 14.46 kg/m^2^hMPa, and it reduced to 33.55 ± 2.20 kg/m^2^hMPa after 120 h, a reduction of 76.68 ± 0.81%. The values are means ± 95% confidence intervals based on two replicates (n = 2). These data are significantly lower than those of the four salt solutions (as shown in Figure 4), probably due to the severe flow resistance resulting from the concentration polarization phenomena when protein molecules are highly rejected [5].

**Figure 8:**
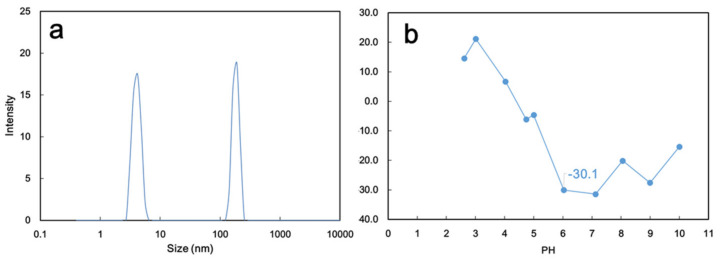
(**a**) Size distribution of BSA in water solution and (**b**) zeta potential of BSA in aqueous solutions at various pH levels, which were adjusted with ammonia or hydrochloric acid solution.

The BSA molecule size at the same concentration (500 ppm) showed a bimodal distribution: 3–5 nm and 140–220 nm (Figure 8a). The smaller molecules represented the protein primary structure [44], and the larger ones may represent the aggregates of the quaternary structure [45]. The smaller BSA molecules accounted for 53%, and this size was smaller than the pore size of the PVDF-PAA (Figure 2e and Table 1). The majority of the primary BSA would have passed through the membrane, and only aggregates would have been retained if the separation was governed by a sieving mechanism. Moreover, the BSA rejection rate would be expected to be 47%. Surprisingly, the BSA rejection rate was as high as 95.5 ± 1.7% (Figure 6), even at various elapsed times (Figure 7). Such a high BSA rejection coefficient suggests that there was another mechanism accounting for the protein filtration behavior. The BSA in the aqueous solution exhibited a zeta potential of −30.1 mV; the solution had a pH value of 6 (Figure 8b). The negatively charged protein molecules were readily rejected by the negatively charged PVDF-PAA top layer, implying that the Donnan exclusion mechanism plays a key role on the BSA protein separation through this membrane.

### 3.3. Performance of Non-Woven/PVDF-PAA-GO Composite Membrane

#### 3.3.1. Permeance and Separation Performance with Salt Solutions

The water permeance through the non-woven/PVDF-PAA-GO composite was 276 kg/m^2^hMPa, which was 12% higher than that through the GO-free composite membrane. The pore size was increased at the expense of the porosity (Table 1) after GO incorporation into the PVDF-PAA layer. Wang et al. and Safarpour et al. found the porosity values increased when GO (less than 0.2 wt%) was added to the PVDF membranes [16,17]. The GO affinity and its hydrophilic groups increased the mass transfer between the solvent and non-solvent during phase inversion, which formed larger pore channels and increased porosity [16]. In this work, the small amount of PAA additive could have delayed the polymer blend’s phase inversion, resulting in a different impact (i.e., a decrease) on porosity.

The permeance values also declined with operating time for the four salt solutions (Figure 3b) using the membrane containing GO. The highest permeance was obtained for the Na_2_SO_4_ solution (164 kg/m^2^hMPa), followed by for the MgSO_4,_ MgCl_2_, and NaCl solutions (110.6, 109.5, and 104.1 kg/m^2^hMPa, respectively, Figure 4). The sequence of the permeance values for the non-woven/PVDF-PAA-GO composite was similar to that of the composite without GO. Again, salt activity played an important role in the water permeation rate, as described in the previous section.

The salt rejection coefficients were stable after 40 min of operation (Figure 5b). Despite the enlarged pore size, the Na_2_SO_4_ was rejected at 24%. The other three salts of MgSO_4_, MgCl_2_, and NaCl were rejected at 11–12% (Figure 6). The salt rejection rates of the GO-containing membrane were higher due to the highly negative zeta potential. Again, the Donnan exclusion mechanism plays a key role in rejecting ions, as the zeta potential of the PVDF-PAA-GO top layer was further reduced to −31 mV from −19 mV of the control PVDF-PAA membrane (Table 1). 

#### 3.3.2. Separation Performance on the BSA Solution

The initial permeance value of the BSA solution through the non-woven/PVDF-PAA-GO membrane was 198.75 ± 39.53 kg/m^2^hMPa and was reduced to 60.82 ± 15.22 Kg/m^2^hMPa after 120 h (Figure 7b), a reduction of 70.08 ± 1.71%. The steady-state mean permeance was 81% higher than that of the membrane without GO (Figure 7a), and there was a smaller flux decline as well. The highly negative zeta potential on the GO-blended membrane reduced protein build-up, thereby minimizing concentration polarization and resulting in higher permeance. 

The BSA showed isolated protein molecules (3–5 nm) and aggregates (140–220 nm, Figure 8a). The rejection of BSA protein molecules was 98.4%, higher than that (95.5%) of the GO-free membrane (Figure 6). Although the pores were enlarged, the surface was even more negative and readily rejected negatively charged BSA molecules through electrostatic repulsion. To further examine the anti-fouling effects, the flux recovery ratio of BSA solution using PVDF-PAA and PVDF-PAA-GO composite membranes was studied. The results confirmed that the PVDF-PAA-GO composite had a flux recovery ratio of 95% at 60 h operation, higher than that of GO-free membrane (85%). The repulsive static force and high hydrophilicity of the GO composite improved the fouling resistance. 

**Figure 9:**
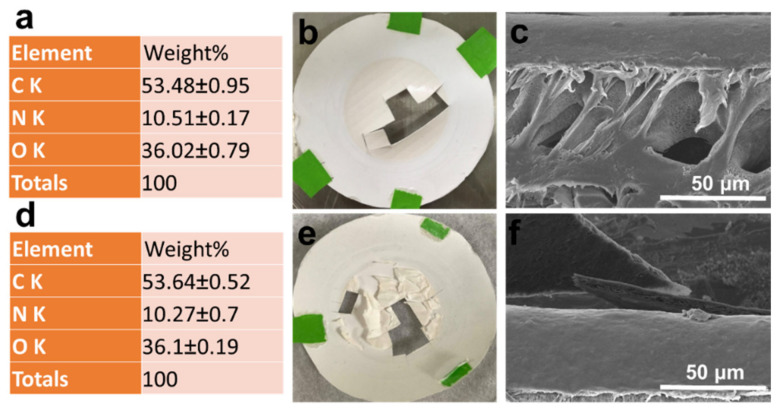
Characterization of non-woven/PVDF-PAA and non-woven/PVDF-PAA-GO membranes after BSA filtration for 120 h: (**a**,**d**) EDX results, (**b**,**e**) photos after freeze-drying, and (**c**,**f**) cross-sections of fouled composite membranes. The values of EDX are mean ± standard deviation based on two replicates (n = 2).

After the BSA filtration process was completed, the fouled membranes were cut off and examined under FESEM micrographs and EDX. Based on EDX analysis, the BSA molecules adsorbed on both membrane surfaces were similar, as shown by the nitrogen contents (Figure 9a,d). Figure 9b,e shows the dried membrane photos after portions were cut off for FESEM/EDX analysis. The PVDF-PAA membrane showed clear cuts (Figure 9b) and strong binding between the membrane and the deposited BSA (Figure 9c). However, the BSA on the PVDF-PAA-GO membrane was delaminated from the membrane surface, which was visible to the naked eye (Figure 9e) and under FESEM observation (Figure 9f). This indicates a good anti-fouling property of the GO composite membrane against protein. The high hydrophilicity and negatively charged surface of the GO-blended PVDF-PAA membrane decreased the interactions with the protein, lessened concentration polarization behavior in the boundary layer adjacent to the surface, and resulted in the higher permeation rate and lesser flux decline than the GO-free membrane.

## 4. Conclusions

In this work, GO was synthesized from exfoliated graphite using the modified Hummer method, redistributed in NMP solvent, and incorporated into the PVDF-PAA blend solution to form a microporous layer on non-woven support. The mean pore size of the GO-free PVDF-PAA membrane was 0.12 μm and was enlarged to 0.16 μm upon GO addition. The zeta potential was −19 mV for PVDF-PAA and reduced to −31 mV for the PVDF-PAA-GO composite membrane. This negative surface charge repelled sodium and magnesium salts and resulted in salt rejection coefficients of 9–28%, even though these ions were smaller than the pores. These salts’ removal was mainly based on the Donnan exclusion mechanism, and the permeance was controlled by salt activity. 

The BSA molecules were present in the primary protein and aggregates, with sizes of 3–5 nm (53%) and 140–220 nm (47%), respectively. The negatively charged BSA (with a zeta potential of −30 mV) was highly rejected using these composite membranes: 95.5% on PVDF-PAA and 98.4% on PVDF-PAA-GO separation layers. Both BSA and membrane were negatively charged, and thereby the protein rejection was very high. In addition, the permeance of the GO composite was 1.8 times higher than that without GO due to weak protein adsorption, which indicates a good anti-fouling property. Overall, the GO composite rendered higher water flux and almost complete BSA rejection. 

## Figures and Tables

**Table 1:** Water contact angle, zeta potential, mean pore size, porosity, and thickness of non-woven PVDF, non-woven/PVDF-PAA, and non-woven/PVDF-PAA-GO composite membranes.

Membrane	Thickness (μm)	Mean Pore Size ^b^ (μm)	Volume Porosity ^d^ (%)	Surface Zeta Potential (mV)	Contact Angle (°)
Non-woven	97	7.13 ± 3.33	-	−3.91 ± 0.58	77.2
Non-woven/PVDF-PAA	97/56 ^a^	0.12 ± 0.02 ^c^	15.6 ^d^	−19.36 ± 0.57	74.9
Non-woven/PVDF-PAA-GO	97/54 ^a^	0.16 ± 0.07 ^c^	28.7 ^d^	−30.88 ± 1.14	59.3

^a^ The numbers represent thicknesses for the non-woven support and top separation layer. ^b^ Data obtained from CFP. ^c^ Data obtained with non-woven support. ^d^ Data obtained without non-woven support.

**Table 2:** Comparison of pure water permeance values with those of various membranes stated in the literature and with those of our composite membranes.

Membrane	Permeance (kg/m^2^hMPa)	Ref.
PVDF-PAA	260	[37]
PVDF/CMC-ZnO (0.05 wt%)	128.9	[40]
Non-woven/PVDF-PAA	203.1	[20]
Non-woven/PVDF-PAA	246	This work
Non-woven/PVDF-PAA-GO	276	This work

**Table 3:** The molar concentration, ionic strength, activity coefficient, and salt activity of various salt solutions, including MgCl_2_, MgSO_4_, NaCl, and Na_2_SO_4_.

Salt	Mole Con. (M)	Ionic Strength (M)	Activity Coefficient	Salt Activity
MgCl_2_	0.01	0.03	0.709	0.021
NaCl	0.034	0.034	0.832	0.028
MgSO_4_	0.017	0.066	0.382	0.025
Na_2_SO_4_	0.006	0.019	0.754	0.014

## Data Availability

The data presented in this work are available on request from the corresponding author.

## Supplementary Materials

The following supporting information can be downloaded at: https://www.mdpi.com/article/10.3390/membranes13010040/s1, Figure S1: Calibration curves of (**a**) NaCl, (**b**) MgCl_2_, (**c**) Na_2_SO_4_, (**d**) MgSO_4_, and (**e**) BSA solutions; Figure S2: (**a**) TEM image of GO in an aqueous solution, (**b**) particle size of the GO in an aqueous solution, (**c**) XRD pattern of commercial graphite powder and GO, (d) Raman spectra of GO dried from in aqueous solution, and (**e**) FTIR spectra of GO dried from an aqueous solution.
