# Supplementary material for: Separation Mechanisms and Anti-Fouling Properties of a Microporous Polyvinylidene Fluoride–Polyacrylic Acid–Graphene Oxide (PVDF-PAA-GO) Composite Membrane with Salt and Protein Solutions
Source: Membranes (Basel). 2022 Dec 28;13(1):40. doi: 10.3390/membranes13010040 (PMC9860620; doi:10.3390/membranes13010040)
Supplement: Supplementary file 1 [file membranes-13-00040-s001.zip › membranes-1997224-supplementary.pdf]

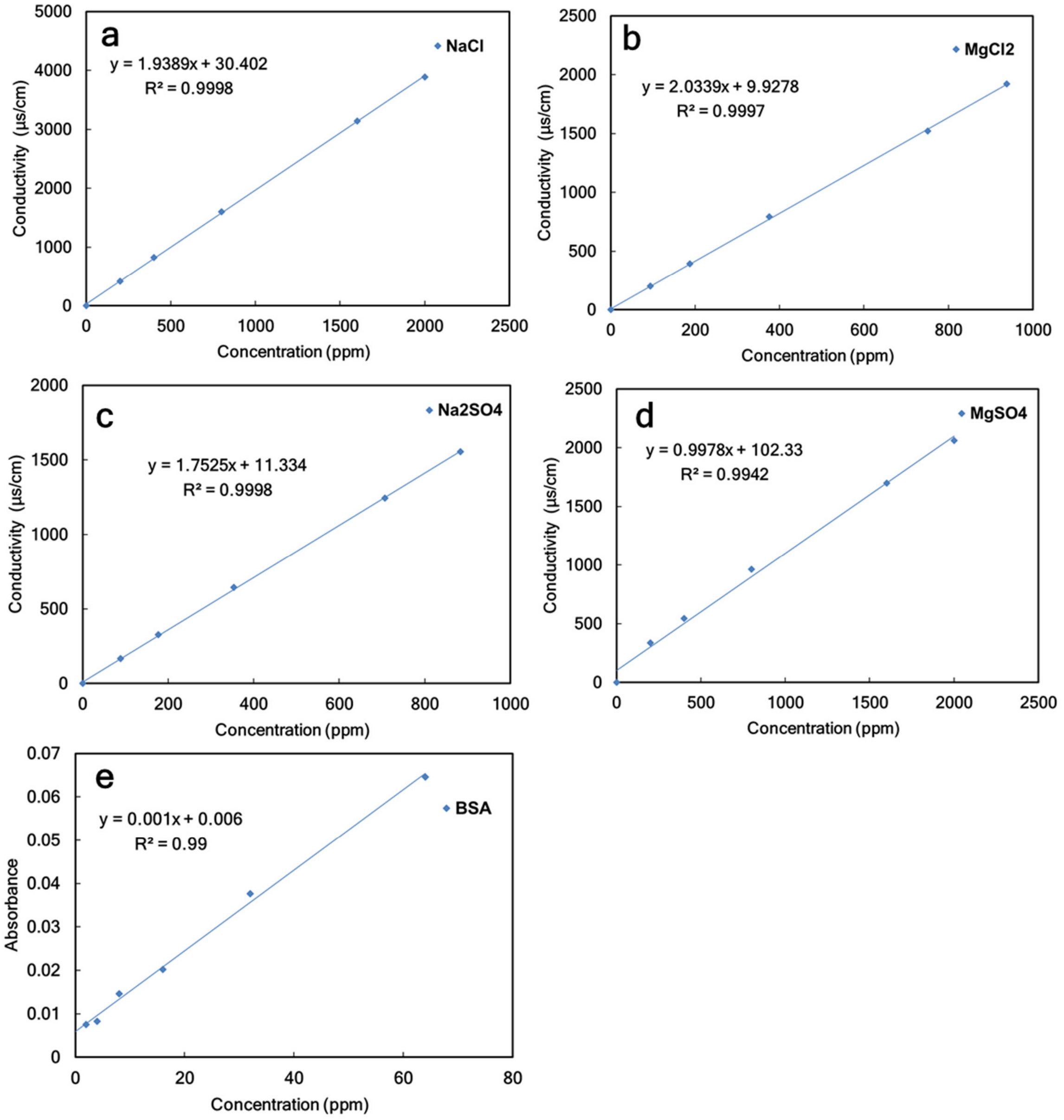

Figure S1. Calibration curves of (**a**) NaCl, (**b**) $MgCl_2$, (**c**) Na_2SO_4, (**d**) $MgSO_4$ and (**e**) BSA solutions.

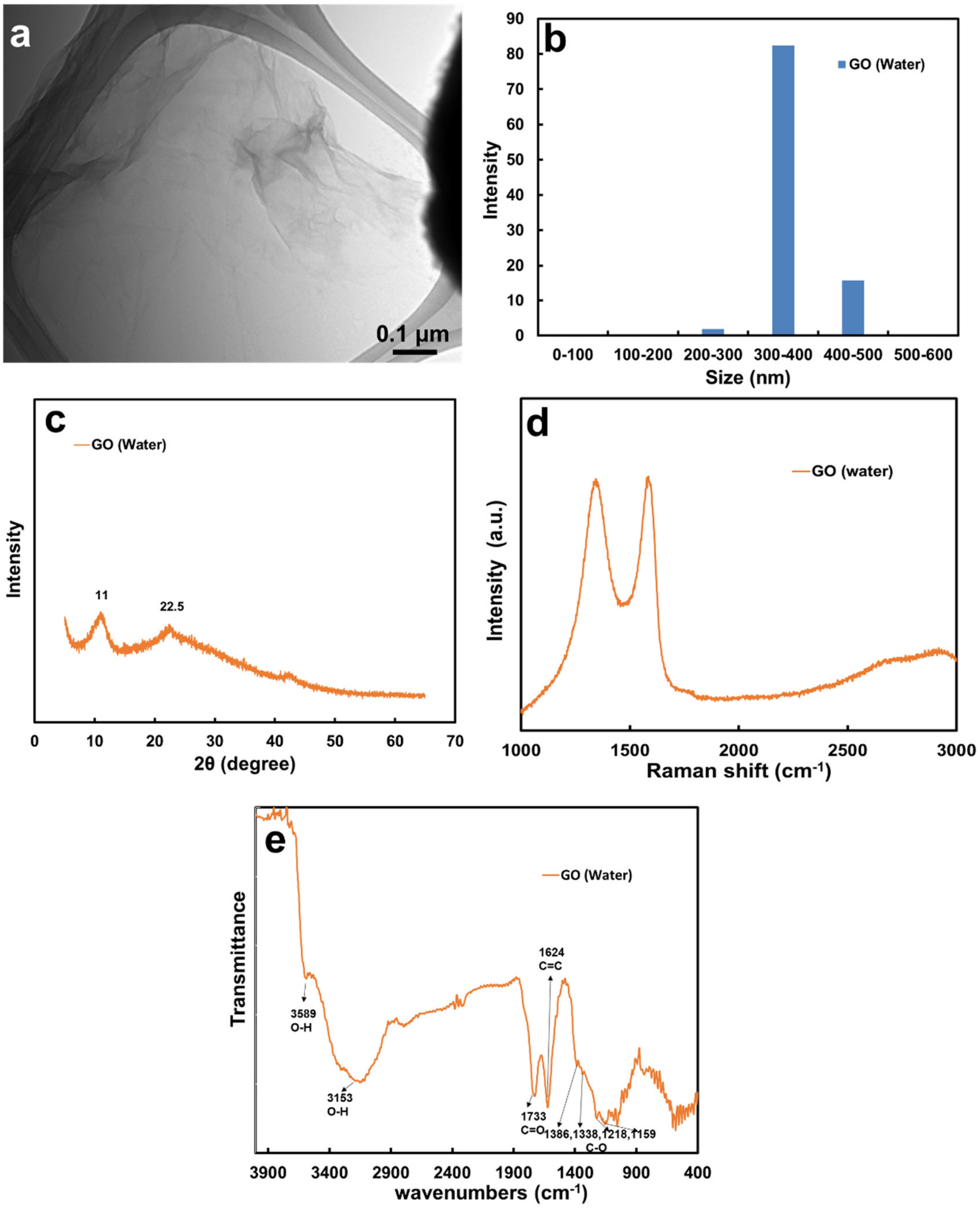

Figure S2. (**a**) TEM image of GO in an aqueous solution, (**b**) particle size of the GO in an aqueous solution, (**c**) XRD pattern of commercial graphite powder and GO, (**d**) Raman spectra of GO dried from in aqueous solution, and (**e**) FTIR spectra of GO dried from an aqueous solution.
